# Pecuniary Affairs of Journal

**Published:** 1857-06

**Authors:** 


*The Pecuniary Affairs of the Journal.*—With the present Number, we inclose bills for arrears of subscription to the *Journal*, as also a circular explanatory of the same Up to this time the *Journal* has not sustained itself because of the want of punctual payment of subscription. Year after year deficits, arising from this source, have been paid by the proprietors, and they now ask those who are behind in their payments to come forward and discharge their obligations. We do not wish to, nor will we, fill our pages hereafter with duns. All reference to such matters shall be by circulars.

We have received numerous letters making inquiries respecting the tardiness of the issue of the present number. We would say to those and all our subscribers, that the pecuniary embarrassments of the *Journal* have made it impossible to avoid such delay. Hereafter, however, we anticipate no such difficulties, and feel warranted in promising its publication, promptly, when due.

We send the present number to all our old subscribers, asking a renewal of subscription upon our published terms. We also send to some who have not been our patrons, and solicit their subscription. Those not wishing to subscribe will please return the No. to us.

